# Diagnostic accuracy of a novel enzyme-linked immunoassay for the detection of IgG and IgG4 against *Strongyloides stercoralis* based on the recombinant antigens NIE/SsIR

**DOI:** 10.1186/s13071-021-04916-x

**Published:** 2021-08-18

**Authors:** Francesca Tamarozzi, Silvia Stefania Longoni, Cristina Mazzi, Sofia Pettene, Antonio Montresor, Siddhartha Mahanty, Zeno Bisoffi, Dora Buonfrate

**Affiliations:** 1 Department of Infectious Tropical Diseases and Microbiology, IRCCS Sacro Cuore Don Calabria Hospital, Verona, Negrar di Valpolicella Italy; 2 University of Ferrara, Ferrara, Italy; 3 Department of Control of Neglected Tropical Diseases, World Health Organization, Geneva, Switzerland; 4 Department of Infectious Diseases, The Peter Doherty Institute for Infection and Immunity, University of Melbourne and The Royal Melbourne Hospital, Melbourne, VIC 3052 Australia; 5 Department of Diagnostics and Public Health, University of Verona, Verona, Italy

**Keywords:** Strongyloides, Strongyloidiasis, ELISA, Recombinant antigen, NIE, SsIR, Accuracy, Diagnostic study

## Abstract

**Background:**

The diagnosis of strongyloidiasis is challenging. Serological tests are acknowledged to have high sensitivity, but issues due to cross-reactions with other parasites, native parasite antigen supply and intrinsic test variability do occur. Assays based on recombinant antigens could represent an improvement. The aim of this study was to assess the sensitivity and specificity of two novel immunoglobulin (Ig)G and IgG4 enzyme-linked immunosorbent assays (ELISAs) based on the recombinant antigens NIE/SsIR for the diagnosis of strongyloidiasis.

**Methods:**

This was a retrospective diagnostic accuracy study. We included serum samples collected from immigrants from strongyloidiasis endemic areas for whom there was a matched result for *Strongyloides stercoralis* on agar plate culture and/or PCR assay, or a positive microscopy for *S. stercoralis* larvae. For the included samples, results were also available from an in-house indirect fluorescent antibody test (IFAT) and a commercial (Bordier ELISA; Bordier Affinity Products SA) ELISA. We excluded: (i) samples with insufficient serum volume; (ii) samples from patients treated with ivermectin in the previous 6 months; and (iii) sera from patients for whom only routine coproparasitology was performed after formol–ether concentration, if negative for *S. stercoralis* larvae. The performance of the novel assays was assessed against: (i) a primary reference standard, with samples classified as negative/positive on the basis of the results of fecal tests; (ii) a composite reference standard (CRS), which also considered patients to be positive who had concordant positive results for the IFAT and Bordier ELISA or with a single “high titer” positive result for the IFAT or Bordier ELISA. Samples with a single positive test, either for the IFAT or Bordier ELISA, at low titer, were considered to be “indeterminate,” and analyses were carried out with and without their inclusion.

**Results:**

When assessed against the primary reference standard, the sensitivities of the IgG and IgG4 ELISAs were 92% (95% confidence interval [CI]: 88–97%) and 81% (95% CI: 74–87%), respectively, and the specificities were 91% (95% CI: 88–95%) and 94% (95% CI: 91–97%), respectively. When tested against the CRS, the IgG ELISA performed best, with 78% sensitivity (95% CI: 72–83%) and 98% specificity (95% CI: 96–100%), when a cut-off of 0.675 was applied and the indeterminate samples were excluded from the analysis.

**Conclusion:**

The NIE-SsIR IgG ELISA demonstrated better accuracy than the IgG4 assay and was deemed promising particularly for serosurveys in endemic areas.

**Graphical abstract:**

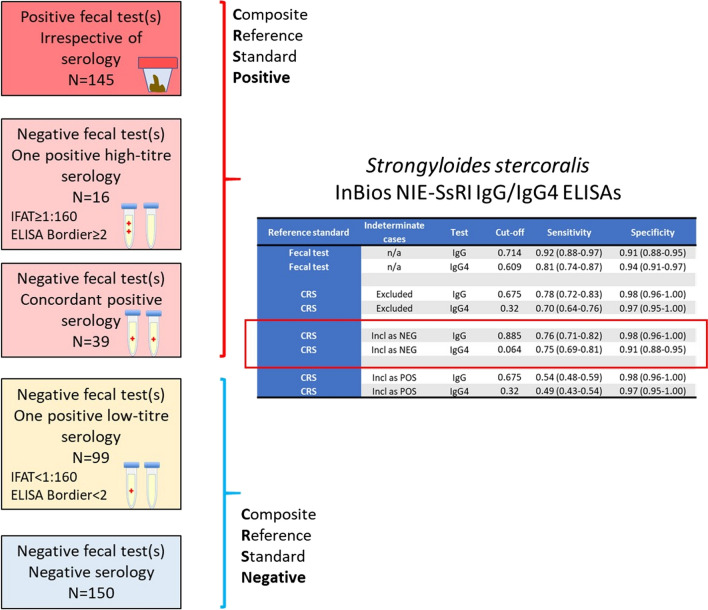

**Supplementary Information:**

The online version contains supplementary material available at 10.1186/s13071-021-04916-x.

## Background

*Strongyloides stercoralis* is a soil-transmitted helminth (STH) which affects about 600 million people in disadvantaged areas of the world [1], mainly in tropical/subtropical areas of South-East Asia, Africa, Western Pacific and Latin America. The infection is transmitted through contact with contaminated soil and perpetuates in the infected individuals due to the continuous re-exposure to the infective source in endemic areas and to a peculiar auto-infective cycle [2]. The latter is caused by larvae produced by the adult female worm in the human intestine that mature into the infective stage in the distal end of the bowel, subsequently penetrating the rectum wall or the perineal skin, leading to chronic infection. Strongyloidiasis often goes unnoticed due to intermittent, non-specific symptoms, and eosinophilia can sometimes be the only indicator of cryptic infection [2]. However, the infection is particularly significant in immunocompromised patients, in whom it can trigger severe disease, characterized by increased larval load (thus, worsening of symptoms) and dissemination of the parasite all over the body. This condition is almost invariably fatal [2].

The first-line treatment (a single dose of 200 µg/kg ivermectin) has an efficacy of around 86% for chronic uncomplicated infection [3]. Treatment of the disseminated disease is more challenging and relies on expert opinion [2].

Despite the potential for harm, the clinical burden and the prevalence of this parasitic infection have long been underestimated, mostly due to inadequacies of diagnostic tests [4]. Indeed, the “traditional” coproparasitological examination has an extremely low sensitivity for *S. stercoralis.* The sensitivity of other stool-based tests, such as agar plate culture (APC), Baermann method and PCR assay, is relatively better, but still unsatisfactory, particularly for low-burden infections [5].

Currently, serology testing is the most sensitive diagnostic method, with sensitivities of 83–92% for commercial enzyme-linked immunosorbent assay (ELISA) kits that use crude larval antigens, and 95% with an in-house immunofluorescence assay (IFAT) [6–8]. The latter detects immunoglobulin (Ig)G antibodies against intact *S. stercoralis* filariform larvae obtained from positive charcoal fecal culture [9]. A previous retrospective study demonstrated good accuracy, with 94.6% (95% conficence interval [CI]: 90.7–98.5%) sensitivity and 87.4% (95% CI: 83.4–91.3%) specificity [6]. An ELISA test used at the study site (*Strongyloides ratti* IgG ELISA) detects antibodies to *Strongyloides* spp. using somatic antigens from larvae of the closely related parasite *S. ratti* [7]. A previous retrospective study estimated its sensitivity and specificity at 90.8% (95% CI: 85.8–95.7%) and 94% (95% CI: 91.2–96.9%), respectively [6].

The need for a constant supply of parasites and the intrinsic variability of the antigenic source are two important limitations of seroassays based on either whole parasites (IFAT) or crude extracts (ELISA) [4].

In a recent World Health Organization meeting [10], seroassays based on recombinant antigens (which do not rely on supplies of larvae) have been identified as the most promising diagnostic method for large-scale use because of the better reproducibility of the results and the commercial availability.

An ELISA assay based on the recombinant *S. stercoralis* NIE antigen has to date showed a sensitivity in the range of 71–84% [6, 11, 12]. Another recombinant antigen, SsIR, has also been developed and evaluated in combination with a luciferase immunoprecipitation system (LIPS) [11, 13], showing excellent results in terms of sensitivity and specificity, both around 97%. Moreover, when the LIPS was used with the combination of NIE and SsIR antigens, there was a further improvement in diagnostic accuracy [13]. However, LIPS technology is not widely available and requires significant capital outlay.

Recently, two ELISA assays based on a combination of NIE and SsRI antigens were developed by InBios (InBios International Inc, Seattle, WA, USA), one for detection of IgG and the second for detection of IgG4 antibodies, for the diagnosis of *S. stercoralis* infection, with the goal of improving the performance of assays based on a single recombinant antigen. The two tests detect, respectively, specific IgG and IgG4 antibodies to *Strongyloides* recombinant antigens NIE and SsIR in serum using an enzymatically amplified sandwich-type immunoassay.

The aim of this study was to assess the sensitivity and specificity of the novel NIE/SsIR IgG and IgG4 ELISAs for the diagnosis of *S. stercoralis* infection using a panel of archived sera and using the results of specific fecal tests or a combination of conventional diagnostic tests (fecal and serology) for the diagnosis of *S. stercoralis* infection as reference.

## Methods

### Study design

This was a single-center, retrospective, diagnostic accuracy study. Sera were retrieved from the “Tropica Biobank” of the Department of Infectious Tropical diseases and Microbiology (DITM) of IRCCS Sacro Cuore Don Calabria hospital, Negrar, Verona, Italy. Results are reported following the STARD (Standards for Reporting Diagnostic Accuracy) guidelines [14].

### Participants and inclusion/exclusion criteria

All available sera in the Tropica Biobank were considered eligible. The inclusion criteria were: (i) serum collected from immigrants from *S. stercoralis* endemic countries (i.e. individuals from Africa, Latin America, South-East Asia and Western Pacific regions); and (ii) serum with a matched result for APC and/or PCR assay for *S. stercoralis* (i.e. the latter test/s done on the same day or ± 30 days from the collection of the serum), or routine coproparasitology after formol–ether concentration if positive for *S. stercoralis* larvae. The exclusion criteria were: (i) unavailable/insufficient quantity of serum; (ii) sera from patients who received treatment with ivermectin in the previous 6 months; and (iii) sera from patients for whom only routine coproparasitology was performed after formol-ether concentration, if negative for *S. stercoralis* larvae.

A list of eligible samples was generated from the electronic database of the biobank, and all consecutive samples meeting the given criteria were included.

### Test methods

#### Fecal tests

Both APC and the PCR assay for *S. stercoralis* are routinely used at the study site. The procedure for APC follows a modified Koga agar plate method. The cultures are incubated up to 6 days and visually inspected on day 2, 4 and 6. On day 6, cultures are flooded with saline and the sediment examined by microscopy. In a previous retrospective study, the sensitivity of the method was 45.4% (95% CI: 30.4–61.1%) [15].

The PCR is a real-time assay based on the method published by Verweij et al. [16]. Briefly, approximately 200 mg of unfixed feces was suspended in 200 µl of phosphate-buffered saline containing 2% polyvinylpolypyrolidone (Sigma-Aldrich Merck KGaA, Darmstadt, Germany) and frozen overnight at − 20 °C. After thawing, the samples were boiled for 10 min at 95 °C. The DNA was then extracted using Magnapure LC.2 extractor (F. Hoffmann-La Roche AG, Basel, Switzerland) following the protocol DNA_I_Blood_Cells_High performance_II, using the DNA isolation kit I (F. Hoffmann-La Roche AG), with a final elution volume of 100 µl. The real-time assay, targeting the small-subunit rRNA gene sequence of *S. stercoralis*, was performed as described. Appropriate positive and negative controls, as well as controls for PCR inhibitors and amplification quality using PhHV-1 control DNA in the same sample reaction, were included in each run. Thermocycling consisted of 40 cycles, and cycle threshold values < 40 were considered positive. The reactions, detection and data analysis were performed with the CFX96 detection system (Bio-Rad, Hercules, CA, USA). In a previous retrospective study the method demonstrated a sensitivity of 56.8% (95% CI: 41.0–71.6%) [15].

#### Serological tests

The following serological tests were used:

i. The new Strongy Detect ™ IgG ELISA and IgG4 ELISA (InBios International, Inc., Seattle, WA, USA). Positive and negative control samples are provided in the kit. The tests were performed according to the manufacturer’s instructions. Interpretation of the results (positive/negative) was based on the analysis of the receiver operating characteristics (ROC) curve, as detailed below.
ii. An in-house IFAT. This IFAT is routinely implemented at DITM for screening and individual diagnosis of strongyloidiasis. A positive result is defined as a titer ≥ 1:20.
iii. The *Strongyloides ratti* IgG ELISA (Bordier ELISA; Bordier Affinity Products SA, Crissier, Switzerland). At DITM, the test is performed as per the manufacturer’s instructions. As the cut-off varies between runs, a normalized optical density (OD) ratio is used to compare the results obtained in different sessions. A ratio ≥ 1 defines positive results. The test is widely available and deployed for routine screening and diagnostic activities across Europe.

### Test procedures

The new InBios ELISA assays were performed by staff of the DITM parasitology laboratory, which is a regional reference laboratory for parasitic infections. Sera were re-coded by staff not directly involved in the laboratory procedures. The laboratory staff were blinded to the results of any fecal and serological tests previously performed. The samples were tested shortly after thawing. For sera already tested using the Bordier ELISA and IFAT at the time of routine analysis, only the new InBios ELISA tests were performed. Conversely, in case results for either routine seroassay were missing, the Bordier ELISA and/or IFAT were carried out as needed.

Data on concomitant infections, assessed as per routine procedure in the parasitology laboratory, were retrieved from the electronic clinical records of DITM. These included routine microscopy after formol–ether concentration of feces for intestinal helminths and ELISA serology for filariasis and schistosomiasis (both assays from Bordier Affinity Products SA).

### Data analysis

#### Reference standards and sera classification

The performances of the new InBios ELISA tests were first assessed against the results of fecal tests only (primary reference standard). For this endpoint, *S. stercoralis* infection was defined based on at least one positive fecal test among standard copromicroscopy of formol–ether concentrated feces, or on positive APC or PCR results.

As the included fecal tests are virtually 100% specific but lack sensitivity, a composite reference standard (CRS) based on the combination of the results of fecal and serological tests was also used to classify patients, as recommended for the evaluation of diagnostic tests in the absence of a gold standard [17]. Patients were classified as positive for *S. stercoralis* infection if they were: (i) positive on at least one fecal test among standard copromicroscopy of formol–ether-concentrated feces, APC or PCR; or (ii) negative on all fecal tests performed, including at least APC or PCR, but concordantly positive by Bordier ELISA and IFAT; or (iii) negative on all fecal tests performed, including at least APC or PCR, and positive only in one routine serology test but at a high titer (≥ 1:160 for IFAT, ≥ 2 for Bordier ELISA). These threshold values were chosen based on the results of a previous study [6]. Negativity for *S. stercoralis* infection was defined in the presence of negative fecal (all performed, including at least APC or PCR) and routine serology (Bordier ELISA and IFAT) tests. Finally, doubtful infection status (indeterminate cases) was defined when there was a negative fecal test (all performed, including at least APC or PCR) and positivity in only one of the two routine seroassays, at a low titer (< 1:160 for IFAT or < 2 for Bordier ELISA).

### Sample size calculation

For sample size calculation, it was assumed that the new InBios ELISAs based on recombinant antigens NIE and SsIR would not have lower sensitivity and specificity than the existing NIE ELISA (75.4 and 89.5%, respectively [6]). For sensitivity, a sample size of 246 was estimated to be required based on a two-sided 95% CI of 0.10 width and test sensitivity of 0.80. For specificity, a sample size of 151 was calculated, assuming a specificity of 0.89 with a 95% CI with width of 0.10. Calculations were carried out using Power Analysis and Sample Size Software (PASS) version 2019 (NCSS Statistical Software, Kaysville, UT USA).

### Statistical analysis

The optimal cut-off for the two index ELISA assays was obtained using the maximum sum of sensitivity and specificity (Youden’s J statistic), determined across all cut-off points of the ROC curve for each endpoint. Cut-off points obtained minimizing the distance to the upper-left corner of the ROC plot (D0-1) were also calculated. Individual samples analyzed with the InBios ELISA tests with OD exceeding the reading limit of the ELISA reader were assigned an OD = 5. Areas under the ROC curve (AUC) were compared by the DeLong test.

Tests results were displayed in contingency tables from which sensitivity and specificity were calculated against the two reference standards: fecal test (primary reference standard) and CRS. When applying the CRS, results obtained from sera of patients with doubtful infection status (i.e. indeterminate cases with negative fecal tests and positive serology at “low titer” in only one of the routine IFAT and Bordier ELISA tests) were first excluded from the analysis, then included while classifying them as either positive or negative.

The association between serology results of the index ELISA assays and presence of other intestinal helminth infections, schistosomiasis or filariasis was assessed using the Chi-square or Fisher Exact tests. Differences between sensitivities and specificities of the two tests were assessed using the McNemar test. Estimations were reported together with the 95% CI. Data analysis was performed using SAS software version 9.4 (SAS Institute, Cary, NC, USA).

## Results

**Fig. 1:**
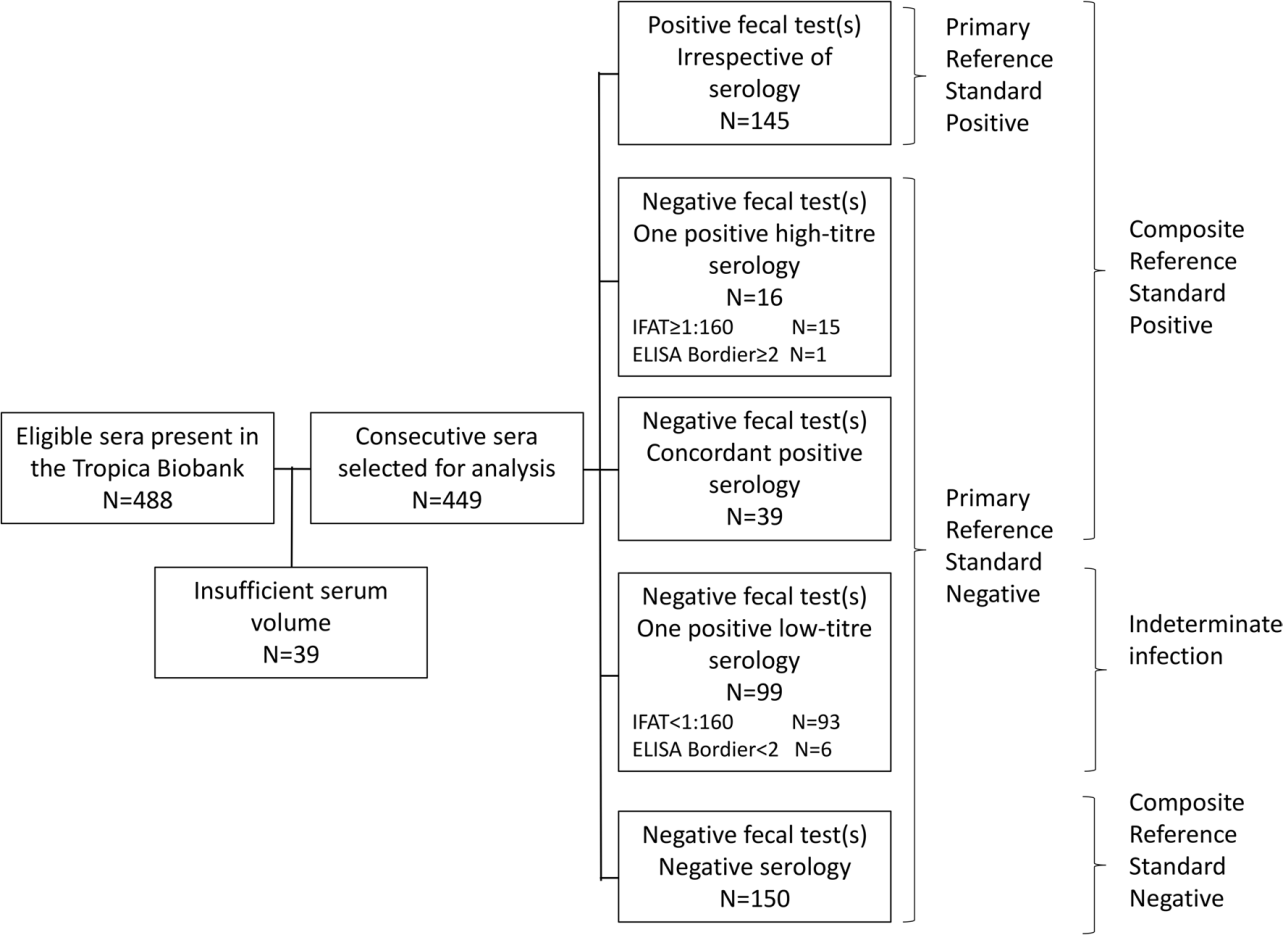
Study flow chart of sample selection.* ELISA* Enzyme-linked immunosorbent assay,* IFAT *indirect fluorescent antibody test

Tests were performed between the 4 and 31 March 2021. The study flow chart of sample selection is reported in Fig. 1.

### Primary reference standard

Of the 449 (32.3%) cases examined, 145 were classified as positive based on at least one positive fecal test: 83 (57.2% of positive samples) had positive APC, 83 (57.2%) had positive stool microscopy and 77 (53.1%) had positive real-time PCR. Table 1 summarizes the results of the two index ELISA tests against this primary reference standard, considering cut-off points obtained by Youden’s J statistic; the results obtained using the cut-off calculated by the D0-1 method are reported in Additional file 1: Table S1.

**Table 1:** Results of of the two NIE/SsIR antigen-based tests against the primary reference standard

ELISA kit	Results of ELISA kit	Fecal tests (*n*)		
		Positive	Negative	Total
InBios Strongy Detect™ IgG ELISA	Positive	135	26	161
	Negative	10	278	288
Total		145	304	449
InBios Strongy Detect™ IgG4 ELISA	Positive	118	18	136
	Negative	27	286	313
Total		145	304	449

ELISA, Enzyme-linked immunosorbent assay, Ig, immunoglobulin

Of note, there were four cases classified as positive on the basis of fecal tests that had concordant negative IFAT and Bordier ELISA results. One of these also tested negative with the new NIE-SsIR; this sample originated from an immunocompetent 2-year-old child. The other three samples were positive for the IgG and IgG4 NIE-SsIR ELISA tests; these samples originated from a patient with tuberculosis, a patient with HTLV-1 infection and a patient with advanced metastatic cancer who developed disseminated strongyloidiasis, respectively.

**Fig. 2:**
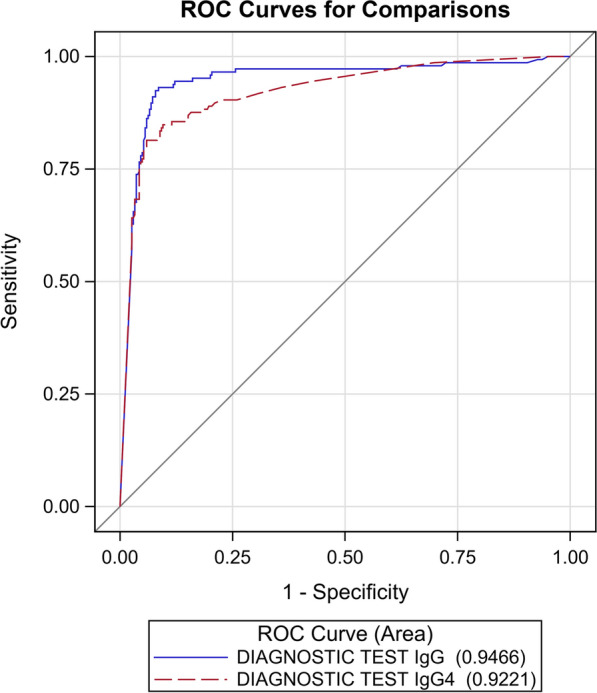
ROC curves for the IgG and IgG4 NIE-SsIR ELISA tests, calculated with the primary reference standard.* Ig* Immunoglobulin,* ROC* receiver operating characteristics

The AUC of the ROC curves (Fig. 2) of the two index tests generated by the results reported in Table 1 was not statistically significantly different (*P* = 0.1210).

### Composite reference standard

The entire panel of 449 sera was classified into 200 positive, 150 negative and 99 indeterminate cases, according to the CRS. First, the accuracy analysis was carried out, excluding the indeterminate cases. The results obtained when applying the cut-off calculated using Youden’s J statistic are reported in Table 2; the results obtained using the cut-off calculated by the D0-1 method are reported in Additional file 1: Table S1.

**Table 2:** Results of the two NIE/SsIR antigen-based tests against the composite reference standard, excluding indeterminate cases

ELISA kit	Results of ELISA kit	Composite reference standard—indeterminate cases excluded (*n*)		
		Positive	Negative	Total
InBios Strongy Detect™ IgG ELISA	Positive	155	3	158
	Negative	45	147	192
Total		200	150	350
InBios Strongy Detect™ IgG4 ELISA	Positive	140	4	144
	Negative	60	146	206
Total		200	150	350

Among cases classified as positives, some had OD values in the InBios ELISAs exceeding the reading limit of the ELISA plate reader: 68 cases for both IgG and IgG4, 23 cases for IgG only and 24 cases for IgG4 only. All of these samples had either positive fecal test results or a concordant “high titer” positive IFAT and Bordier ELISA. In addition, one case with an OD reading for IgG4 that exceeded the upper limit was observed in the strongyloidiasis-negative group; in this case, the IgG ELISA was also positive (OD 1.37). This sample originated from a patient who had been diagnosed with strongyloidiasis (positive APC and IFAT 1:20) and treated with ivermectin 7 years previously. At the time the sample included in this study was collected, the patient had come back for itching, had a normal eosinophil count and was negative for *S. stercoralis* by IFAT, APC and PCR

**Fig. 3:**
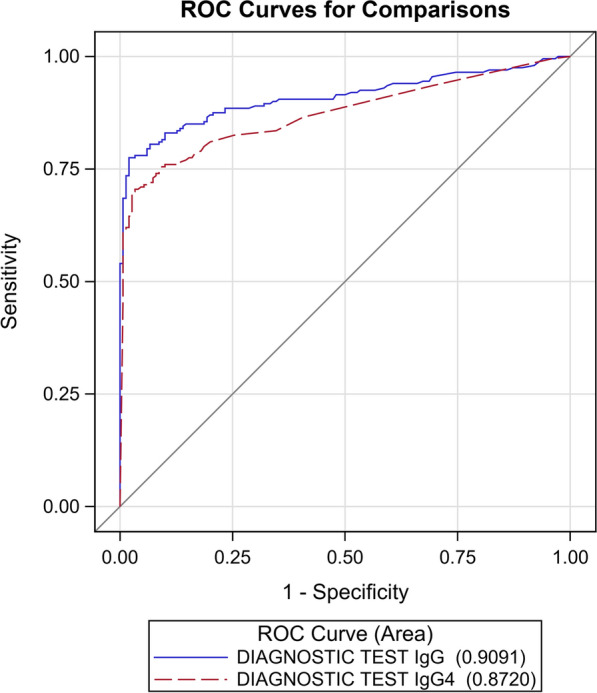
ROC curves for the IgG and IgG4 NIE-SsIR ELISA tests, calculated with the composite reference standard, excluding indeterminate cases

The AUC of the ROC curves (Fig. 3) generated by the results reported in Table 2 was statistically significantly different (*P* = 0.035).

We then assessed the index ELISA cut-off and sensitivity and specificity against a CRS where indeterminate cases were classified as negative cases. The results obtained when applying the cut-off calculated using the Youden’s J statistics are reported in Table 3; the results obtained using the cut-off calculated by the D0-1 method are reported in Additional file 1: Table S1.

**Table 3:** Results of the two NIE-SsIR antigen-based tests against the CRS, indeterminate cases were classified as negative cases

ELISA kit	Results of ELISA kit	Composite reference standard—indeterminate cases included as negative cases (*n*)		
		Positive	Negative	Totals
InBios Strongy Detect™ IgG ELISA	Positive	153	5	158
	Negative	47	244	291
Total		200	249	449
InBios Strongy Detect™ IgG4 ELISA	Positive	150	22	172
	Negative	50	227	277
Total		200	249	449

**Fig. 4:**
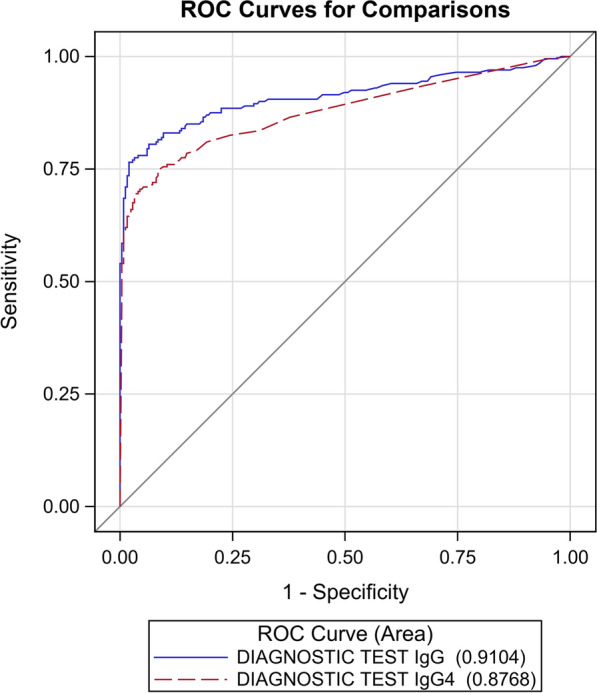
ROC curves for the IgG and IgG4 NIE-SsIR ELISA tests, calculated with the composite reference standard. Indeterminate cases were classified as negative cases

The AUC of the related ROC curves (Fig. 4) was significantly different (*P* = 0.0389).

Finally, the sensitivity and specificity of the index ELISA cut-off were assessed against a CRS where indeterminate cases were classified as positive. The results obtained when applying the cut-off calculated using the Youden’s J statistics are reported in Table 4; the results obtained using the cut-off calculated by the D0-1 method are reported in Additional file 1: Table S1.

**Table 4:** Results of the two NIE-SsIR antigen-based tests against the CRS, indeterminate cases were classified as positive cases

ELISA kit	Results of ELISA kit	Composite reference standard—indeterminate cases included as positive cases (*n*)		
		Positive	Negative	Total
InBios Strongy Detect™ IgG ELISA	Positive	160	3	163
	Negative	139	147	291
Total		299	150	449
InBios Strongy Detect™ IgG4 ELISA	Positive	146	4	150
	Negative	153	146	277
Total		299	150	449

**Fig. 5:**
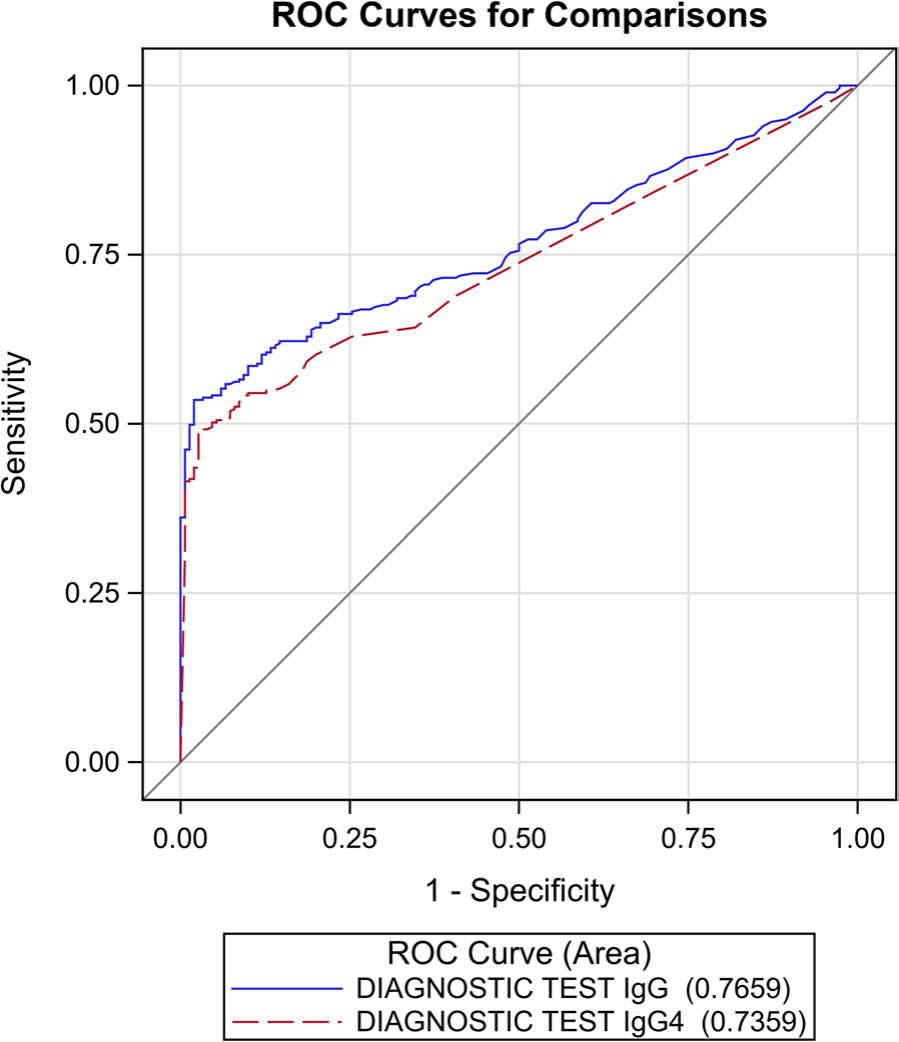
ROC curves for the IgG and IgG4 NIE-SsIR ELISA tests, calculated with the composite reference standard. Indeterminate cases were classified as positive cases

The AUC of the ROC curves (Fig. 5) of the two index tests generated by the results reported in Table 4 was not statistically significantly different (*P* = 0.1728).

The cut-off values and the sensitivity and specificity calculated based on primary and composite reference standards and cut-off points generated using the Youdens’ J statistics, including when indeterminate cases were included as positive cases in the analysis, are summarized in Table 5; the cut-off and accuracy results calculated with the D0-1 method are reported in Additional File 1: Table S1.

**Table 5:** Cut-off values, sensitivity and specificity of the index InBios ELISA tests using the primary (fecal test) and secondary (CRS) reference standards

Reference standard	Indeterminate cases	Test	Cut-off value	Sensitivity (95% CI)	*P*^a^	Specificity (95% CI)	*P*^a^
Primary—fecal test	n/a	IgG	0.714	0.93 (0.88–0.97)	< 0.0001	0.91 (0.88–0.95)	0.0325
Primary—fecal test	n/a	IgG4	0.609	0.81 (0.74–0.87)		0.94 (0.91–0.97)	
Secondary—CRS	Excluded	IgG	0.675	0.78 (0.72–0.83)	0.0011	0.98 (0.96–1.00)	0.6547
Secondary—CRS	Excluded	IgG4	0.32	0.70 (0.64–0.76)		0.97 (0.95–1.00)	
Secondary—CRS	Included as negative cases	IgG	0.885	0.76 (0.71–0.82)	0.5316	0.98 (0.96–1.00)	0.0004
Secondary—CRS	Included as negative cases	IgG4	0.064	0.75 (0.69–0.81)		0.91 (0.88–0.95)	
Secondary—CRS	Included as positive cases	IgG	0.675	0.54 (0.48–0.59)	0.0082	0.98 (0.96–1.00)	0.6547
Secondary—CRS	Included as positive cases	IgG4	0.32	0.49 (0.43–0.54)		0.97 (0.95–1.00)	

CI, Confidence interval; CRS, composite reference standard, n/a, not available

^a^*P*-values comparing sensitivities and specificities were determined by McNemar’s test

#### Association between positive serology for* S. stercoralis* and concurrent helminth infections

In the whole cohort, 35 patients had one or more intestinal helminth infections, including hookworm (*n* = 22), *Hymenolepis nana* (*n* = 6), *Trichuris trichiura* (*n* = 7), *Dicrocoelium dendriticum* (*n* = 2), *Strongyloides fuelleborni* (*n* = 2), *Trichostrongylus* spp. (*n* = 2), *Ascaris lumbricoides* (*n* = 1) and *Schistosoma* spp. (*n* = 19 with eggs in stool). No significant association was found between the results of the IgG and IgG4 ELISA and concomitant infection with intestinal helminths, in neither the strongyloidiasis-negative or the strongyloidiasis-indeterminate group, or the two groups combined. Significant associations with *Schistosoma* infection and with serology for schistosomiasis (data available for 303 patients) were found only for the IgG4 ELISA in the strongyloidiasis-negative group (*P* = 0.0042 for eggs in stool and *P* = 0.0157 for anti-*Schistosoma* ELISA), and in the strongyloidiasis-negative + indeterminate groups combined (*P* = 0.0162 for eggs in stool and *P* = 0.0517 for anti-*Schistosoma* ELISA). Of note, all *Schistosoma*-positive samples originated from migrants from sub-Saharan Africa. A total of 234 patients had records of a routine ELISA serology exam for filariasis; no significant association was found between filarial serology and both IgG and IgG4 ELISA in the strongyloidiasis-negative or the strongyloidiasis-indeterminate groups, or the two groups combined.

Overall, similar results were found when the same analysis was applied to the routine serology tests for strongyloidiasis by IFAT and Bordier ELISA. No association was found between results of these tests and the presence of intestinal helminth infections in the strongyloidiasis-negative or the strongyloidiasis-indeterminate groups, or the two groups combined. The results of both routine seroassays were significantly associated with *Schistosoma* infection in the strongyloidiasis-indeterminate group (*P* = 0.0027 for both assays) and the *Strongyloides*-negative + indeterminate combined group (*P* = 0.0471 for IFAT and *P* = 0.0086 for Bordier ELISA). When schistosomiasis was assessed with the ELISA, significant associations with IFAT and Bordier ELISA results were found with the *Strongyloides*-indeteminate (*P* = 0.0153 both IFAT and Bordier ELISA) group and with the *Strongyloides*-negative + indeterminate group combined (*P* = 0.0248 Bordier ELISA).

No significant association was also found between IFAT and Bordier ELISA results and filariasis serology in the strongyloidiasis-negative and the strongyloidiasis-indeterminate groups.

Results of the association between positive serology for *S. stercoralis* and concurrent helminth infections are summarized in Additional file 2: Table S2.

## Discussion

In this study, we evaluated two novel ELISAs based on the recombinant antigens NIE and SsIR using samples collected from a cohort of patients from *S. stercoralis* endemic areas, representative of people who would routinely be screened for strongyloidiasis and other parasitic infections in our setting. Due to the lack of a diagnostic gold standard for *S. stercoralis* infection [5], the two assays were evaluated not only against the results of fecal tests (primary reference standard), but also against a CRS using a panel of sera from patients with negative fecal tests results and variable results on routine serology assays performed at DITM. As expected, sensitivity was higher and specificity lower when using the primary reference standard than the CRS. For the latter analysis, we first excluded “indeterminate cases,” i.e. those with a single “low titer” positive routine serological test (IFAT or Bordier ELISA) result, with the aim of reducing the possible misclassification of positive and negative results. We then included the indeterminate cases so that the cohort could mirror real-life situations, where cases of uncertain classification can occur and cane be interpreted as positive or negative. Our results showed that when indeterminate cases were classified as negative, the accuracy of the tests was similar to that obtained when the indeterminate cases were not included in the CRS. This result indicates that classifying indeterminate results as negative was appropriate in terms of setting up the CRS; therefore, here we focus our discussion of the assays’ performance by referring to the results of this approach when addressing the CRS.

The AUC was similar for the two ELISAs when considering the primary reference standard, although sensitivity was significantly higher for IgG (93%; 95% CI: 88–97%) than for IgG4 (81%; 95% CI: 74–87%): *P* < 0.0001.

With the CRS, the AUC was instead statistically different for the two assays, favoring the IgG ELISA, with this assay showing excellent specificity (98%; 95% CI: 96–100%) and good sensitivity (76%, 95% CI: 71–82%) at a cut-off of 0.88 OD. Sensitivity of the IgG4 ELISA was similar to that of the IgG ELISA (*P* = 0.5316), but the specificity of the former was significantly lower (91%; 95% CI: 88–95%, *P* = 0.0004) and, in addition, this IgG4-detecting test showed a significant association with parasitological and serological diagnosis of schistosomiasis that was not shown by the IgG ELISA.

Overall, sensitivity of the two tests seems to be in line with that of the previous NIE-ELISA, while the specificity of the NIE-SsIR-IgG is improved compared with that of the NIE-ELISA [6, 12]. Other serological assays (namely our in-house IFAT, Bordier ELISA, IVD [in vitro diagnostic] ELISA) previously demonstrated higher sensitivity than these two assays [6, 7]; however, it must be noted that the two recombinant ELISAs detected three cases with positive fecal tests but with negative results to the other serological tests; these three cases were in patients with impaired immunity; hence it would be worthwhile to further explore this finding in a larger cohort of immunosuppressed patients, for whom other serological assays may have reduced sensitivity [18, 19].

The choice of tests with different sensitivity and specificity depends on the setting where the tests need to be applied and the diagnostic purpose. In the non-endemic setting, especially at the hospital level, test sensitivity would be prized over specificity, as it would be preferable to treat a falsely-positive patient with ivermectin, which is a well-tolerated and safe drug, than miss cases of this potentially life-threatening infection. In contrast, a high test specificity would be preferred in the context of control programs in endemic areas, where co-infections represent an issue when the prevalence and impact of control activities need to be assessed and the whole target population will receive the intervention(s) in any case, irrespective of the positive or negative result of the test [10, 20]. As mentioned, these programs entail testing large numbers of people; hence, in principle, ELISAs based on recombinant antigens, such as the one evaluated here, would be advantageous over those based on crude antigens on the basis of high reproducibility and supply capacity. Field studies should be carried out using the recombinant-based InBios ELISA assays to confirm their good performance in that setting. Another aspect that would encourage the deployment of these assays in the field is the possibility of performing the test on serum obtained from dried blood spots collected on filter paper, which eases sample collection and transport. Previous studies demonstrated that this is possible for the NIE-ELISA [21] and other serological assays [22], encouraging the evaluation of the NIE-SsIR ELISA tests with this material. Finally, other serological tests have also been demonstrated to be useful for post-treatment monitoring, both in the non-endemic (at individual level) [23, 24] and endemic (at population level) [25] settings. This aspect requires evaluation of these new tests as well in order to frame their usefulness both in the clinical and control program setting to reflect presence of ongoing infection.

The main limitation of this study is its retrospective design. While it was possible to review the clinical files of a few patients with unexpected results, we could not systematically collect clinical data from the entire cohort. In addition, as examinations for concurrent parasitic infections were not performed for all patients, the results of the association with serological test results for strongyloidiasis should be taken with caution and confirmed following a systematic approach. Moreover, the patients did not all undergo the same fecal tests, although we included only samples on which PCR and/or APC were performed, which have comparable accuracy, carried out at a matching time point. One of the major strengths of this study is that the cohort was representative of patients for whom these tests would be used in our non-endemic setting, making our results relevant for the evaluation of the performance of the index ELISA assays in clinical practice. Another strength is the use of a CRS, which permits a robust estimation of the accuracy of a test in the absence of a diagnostic gold standard [17]. However; consideration must be given to the fact that the CRS is still an imperfect tool and that it is possible that the values of sensitivity and specificity of the NIE-SsIR IgG and IgG4 ELISAs actually lay between those found with the primary reference standard and the CRS.

## Conclusions

In conclusion, the NIE-SsIR IgG ELISA demonstrated better accuracy than the IgG4-detecting assay and the previous NIE-ELISA [6, 12]. The sensitivity of the IgG ELISA assay was lower than that demonstrated previously for other serological assays [6, 12], but its specificity was excellent. These characteristics, in addition to possible large-scale production and reproducibility, make the test promising for serosurveys in the field, also in the context of control programs. Further studies should evaluate its use in an endemic setting, preferably also on dried blood spots. In addition, performance for post-treatment monitoring should be assessed both in endemic and non-endemic settings.

## Supplementary Information

**Additional file 1: Table S1.** Cut-off values calculated by the D0-1 method.

**Additional file 2: Table S2.** Results of the association between positive serology for *S. stercoralis* and concurrent helminth infections.

## Data Availability

The raw data will be published in Zenodo upon acceptance for publication of the present article.

## Abbreviations

APC: Agar plate culture
AUC: Area under curve
CRS: Composite reference standard
DITM: Department of Infectious Tropical Diseases and Microbiology
ELISA: Enzyme-linked immunosorbent assay
IFAT: Indirect fluorescent antibody test
LIPS: Luciferase immunoprecipitation system
OD: Optical density
ROC: Receiver operating characteristics
STH: Soil-transmitted helminthes
