# Supplementary material for: Diagnostic accuracy of a novel enzyme-linked immunoassay for the detection of IgG and IgG4 against Strongyloides stercoralis based on the recombinant antigens NIE/SsIR
Source: Parasit Vectors. 2021 Aug 18;14:412. doi: 10.1186/s13071-021-04916-x (PMC8375122; doi:10.1186/s13071-021-04916-x)
Supplement: Supplementary file 1 — Additional file 1: Table S1. Cut-off values calculated by the D0-1 method. [file 13071_2021_4916_MOESM1_ESM.docx]

| **Reference standard** | **Indeterminate**  **cases** | **Test** | **Cut-off** | **Sensitivity** | **Specificity** |
| --- | --- | --- | --- | --- | --- |
| Faecal test only | n/a | IgG | 0,885 | 0,924 (0,881-0,967) | 0,921 (0,891-0,951) |
| Faecal test only | n/a | IgG4 | 0,263 | 0,848 (0,790-0,907) | 0,905 (0,872-0,938) |
| Composite Ref Standard | Excluded | IgG | 0,257 | 0,830 (0,778-0,882) | 0,900 (0,852-0,948) |
| Composite Ref Standard | Excluded | IgG4 | 0,059 | 0,760 (0,701-0,819) | 0,900 (0,852-0,948) |
| Composite Ref Standard | Incl as NEG | IgG | 0,257 | 0,830 (0,778-0,882) | 0,904 (0,867-0,940) |
| Composite Ref Standard | Incl as NEG | IgG4 | 0,044 | 0,785 (0,728-0,842) | 0,851 (0,807-0,896) |
|  |  |  |  |  |  |
| Composite Ref Standard | Incl as POS | IgG | 0,152 | 0,622 (0,567-0,677) | 0,853 (0,797-0,910) |
| Composite Ref Standard | Incl as POS | IgG4 | 0,041 | 0,602 (0,546-0,657) | 0,800 (0,736-0,864) |
