# Supplementary material for: Diagnostic accuracy of a novel enzyme-linked immunoassay for the detection of IgG and IgG4 against Strongyloides stercoralis based on the recombinant antigens NIE/SsIR
Source: Parasit Vectors. 2021 Aug 18;14:412. doi: 10.1186/s13071-021-04916-x (PMC8375122; doi:10.1186/s13071-021-04916-x)
Supplement: Supplementary file 2 — Additional file 2: Table S2. Results of the association between positive serology for S. stercoralis and concurrent helminth infections. [file 13071_2021_4916_MOESM2_ESM.docx]

| Other parasitosis | Ss positive CRS  p-value | Ss indeterminate  p-value | Ss negative  p-value | SS negative + indeterminate  p-value |
| --- | --- | --- | --- | --- |
| InBios IgG ELISA | | | | |
| Intestinal helminths | 0.2273 | 1.0000 | 1.0000 | 1.0000 |
| Schistosoma spp eggs | 0.7317 | 1.0000 | 1.0000 | 1.0000 |
| Schistosoma serology | 0.4797 | 1.0000 | 1.0000 | 1.0000 |
| Filaria  serology | 0.0063 | 0.0882 | 1.0000 | 0.1835 |
| InBios IgG4 ELISA | | | | |
| Intestinal helminths | 0.3467 | 1.000 | 0.4181 | 0.4801 |
| Schistosoma spp eggs | 1.0000 | 1.0000 | 0.0042 | 0.0162 |
| Schistosoma serology | 0.5035 | 1.0000 | 0.0157 | 0.0517 |
| Filaria  serology | 0.0269 | 1.0000 | 1.0000 | 1.0000 |
| IFAT | | | | |
| Intestinal helminths | 0.3552 | 1.0000 | - | 0.2600 |
| Schistosoma spp eggs | 0.4290 | 0.0027 | - | 0.0471 |
| Schistosoma serology | 1.000 | 0.0153 | - | 0.1757 |
| Filaria  serology | 0.6197 | 0.1710 | - | 0.2002 |
| Bordier ELISA | | | | |
| Intestinal helminths | 0.1277 | 1.0000 | - | 1.0000 |
| Schistosomia spp eggs | 1.0000 | 0.0027 | - | 0.0086 |
| Schistosoma serology | 1.0000 | 0.0153 | - | 0.0248 |
| Filaria  serology | 0.0088 | 0.1710 | - | 0.2376 |

Level of significance (p-value, Chi-square test) of the difference in % positive samples for each strongyloidiasis serological test in patients with and without concurrent parasitic infections, within each infection status group. CRS=composite reference standard.
